# Reliability of non-contact tongue diagnosis for Sjögren's syndrome using machine learning method

**DOI:** 10.1038/s41598-023-27764-4

**Published:** 2023-01-24

**Authors:** Keigo Noguchi, Ichiro Saito, Takao Namiki, Yuichiro Yoshimura, Toshiya Nakaguchi

**Affiliations:** 1 Graduate School of Science and Technology, Chiba University, Chiba, Japan; 2 Department of Pathology, Tsurumi University School of Dental Medicine, Yokohama, Japan; 3 Department of Japanese-Oriental (Kampo) Medicine, Graduate School of Medicine, Chiba University, Chiba, Japan; 4 School of Engineering, Chukyo University, Nagoya, Japan; 5 Center for Frontier Medical Engineering, Chiba University, Chiba, Japan

**Keywords:** Biomedical engineering, Scientific data

## Abstract

Sjögren's syndrome (SS) is an autoimmune disease characterized by dry mouth. The cause of SS is unknown, and its diverse symptoms make diagnosis difficult. The Saxon test, an intraoral examination, is used as the primary diagnostic method for SS, however, the risk of salivary infection is problematic. Therefore, we investigate the possibility of diagnosing SS by non-contact and imaging observation of the tongue surface. In this study, we obtained tongue photographs of 60 patients at the Tsurumi University School of Dentistry outpatient clinic to clarify the relationship between the features of the tongue and SS. We divided the tongue into four regions, and the color of each region was transformed into CIE1976L*a*b* space and statistically analyzed. To clarify experimentally the possibility of SS diagnosis using tongue color, we employed three machine-learning models: logistic regression, support vector machine, and random forest. In addition, we constructed diagnostic prediction models based on the Bagging and Stacking methods combined with three machine-learning models for comparative evaluation. This analysis used dimensionality compression by principal component analysis to eliminate redundancy in tongue color information. We found a significant difference between the a* value of the rear part of the tongue and the b* value of the middle part of the tongue in SS and non-SS patients. In addition to the principal component scores of tongue color, the support vector machine was trained using age, and achieved high accuracy (71.3%) and specificity (78.1%). The results indicate that the prediction of SS diagnosis by tongue color reaches a level comparable to machine learning models trained using the Saxon test. This is the first study using machine learning to predict SS diagnosis by non-contact tongue observation. Our proposed method can potentially support early SS detection simply and conveniently, eliminating the risk of infection at diagnosis, and it should be validated and optimized in clinical practice.

## Introduction

Sjögren's syndrome (SS) is an autoimmune disease characterized by dry mouth and dry eyes. It is believed to be caused by a complex relationship between immune abnormalities, female hormone secretion, and heredity; however, the exact cause of this disease is yet to be clarified. Multiple clinical tests have been employed for the diagnosis of SS. One of the commonly used oral examinations is the Saxon test, and it involves the quantitative observation of saliva to determine oral dryness. However, in recent years, the presence of the COVID-19 virus in saliva has made it difficult to conduct saliva tests for the evaluation of oral conditions. Therefore, it is necessary to develop an objective method for evaluating oral conditions.

The most common symptoms of SS include dry mouth, pain in the tongue, fissures on the tongue, and discoloration of the tongue due to oral candidiasis^1^. Moreover, dermoscopic studies have revealed that differences in the appearance of the structure and color of the tongue surface may be important markers for the diagnosis of SS^2^. The features of the tongue, such as color, gloss, and shape provide clinically critical diagnostic clues for the diagnosis of several other diseases^3,4^.

Recent studies have reported the promising potential of machine learning methods in developing several bioinformatics tools^5,6^ and application for analyzing tongue images^7^. The use of imaging devices for the diagnosis of diseases is a simple and quick approach, and may be appropriate as a screening test for various diseases. In a previous study, we developed a tongue image analysis system (TIAS) that can be used for computer-aided tongue diagnosis based on the color of the tongue^8,9^. The essential characteristic of the tongue imaging method of TIAS is the exclusion of the influence of external light by utilizing an integrating sphere to achieve an evenly distributed light intensity. Further, the TIAS can remove the gloss of the tongue surface from its images to stabilize the color of the tongue surface and the coating of the tongue.

Studies predicting the diagnosis of SS from objective information have been very rare. Jesper et al.^10^ classified SS patients based on routinely recorded primary care data. Using the LR and RF models for machine learning, they found that the LR model had an accuracy of 0.82 and the RF model 0.84, respectively. Although the LR and RF models achieved high prediction accuracy, they are not applicable to immediate medical care because they use routinely recorded primary care data as input information, limiting the patients to whom they can be applied.

In this study, we investigated the possibility of diagnosing SS via the machine learning classification of tongue images obtained by TIAS. The results were compared to the classification results of Saxon test, which is a diagnostic criterion for SS, to determine if the method proposed in this study can be used as an alternative method for diagnosing SS during the COVID-19 pandemic.

## Materials and methods

### Tongue image analyzing system (TIAS)

**Figure 1:**
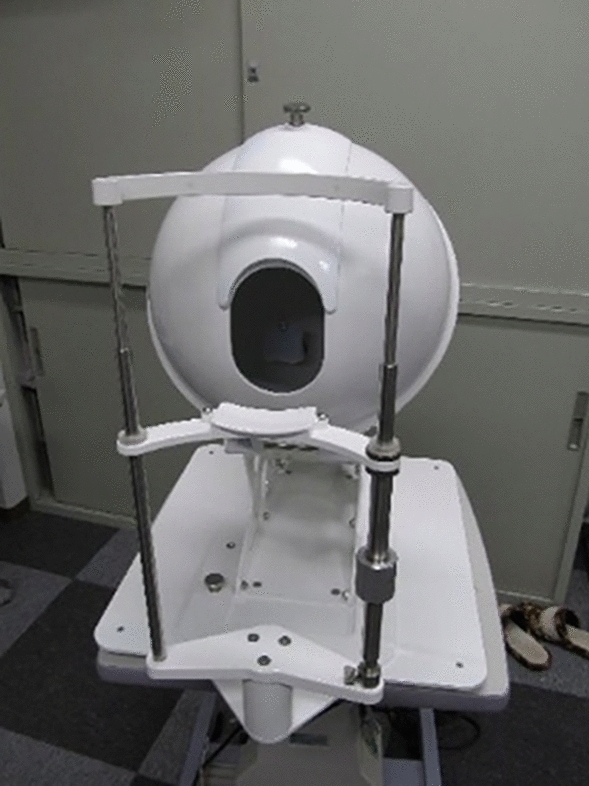
Image of the Tongue Image Analyzing System (TIAS). The TIAS used in this study equipped with a chin rest and a forehead rest for fixing the face of the subject.

TIAS is a photographic device used for capturing the image of the tongue, and it is equipped with a diffused light source for recording the state of the tongue surface. The TIAS used in this study consists of a chin rest and a forehead rest for fixing the face of the subject. To capture the images of the tongue, first, the camera and light source were calibrated using a color checker. The color checker used to calibrate the camera and light source was X-Rite Color Checker (formerly known as Munsell). By photographing the known 24 colors of the color checker, a conversion matrix from the RGB color space of the camera to the XYZ color space is created by multiple regression analysis. The conversion to L*a*b* color space uses the conversion formula specified by CIE. Subsequently, the tongues of the patients were captured 10 times per second for 20 s, and a total of 200 images (1024*1280 pixels) were acquired. From the 200 images, the operator selected one image that was immediately after the tongue sticking out and fully exposed for analysis. Lastly, the captured RGB tongue images were converted to CIE1976L*a*b* based on the conversion matrix estimated from the color charts using the multiple regression method. CIE1976L*a*b* color space is device-independent, and the amount of change in each value is equivalent to the amount of change between the stimulus and human vision. The image of the TIAS used in this study is shown in Fig. 1.

### Tongue color extraction

To obtain the tongue color in the same area each time, the shape of the tongue was defined by manually determining five points along the contour of the tongue. Subsequently, four areas of the tongue were defined with fixed proportions: (1) tongue edge, (2) tongue posterior, (3) tongue middle, and (4) tongue apex. The defined area was a circle with a radius of 10 pixels, and the average color of each area was estimated.

**Figure 2:**
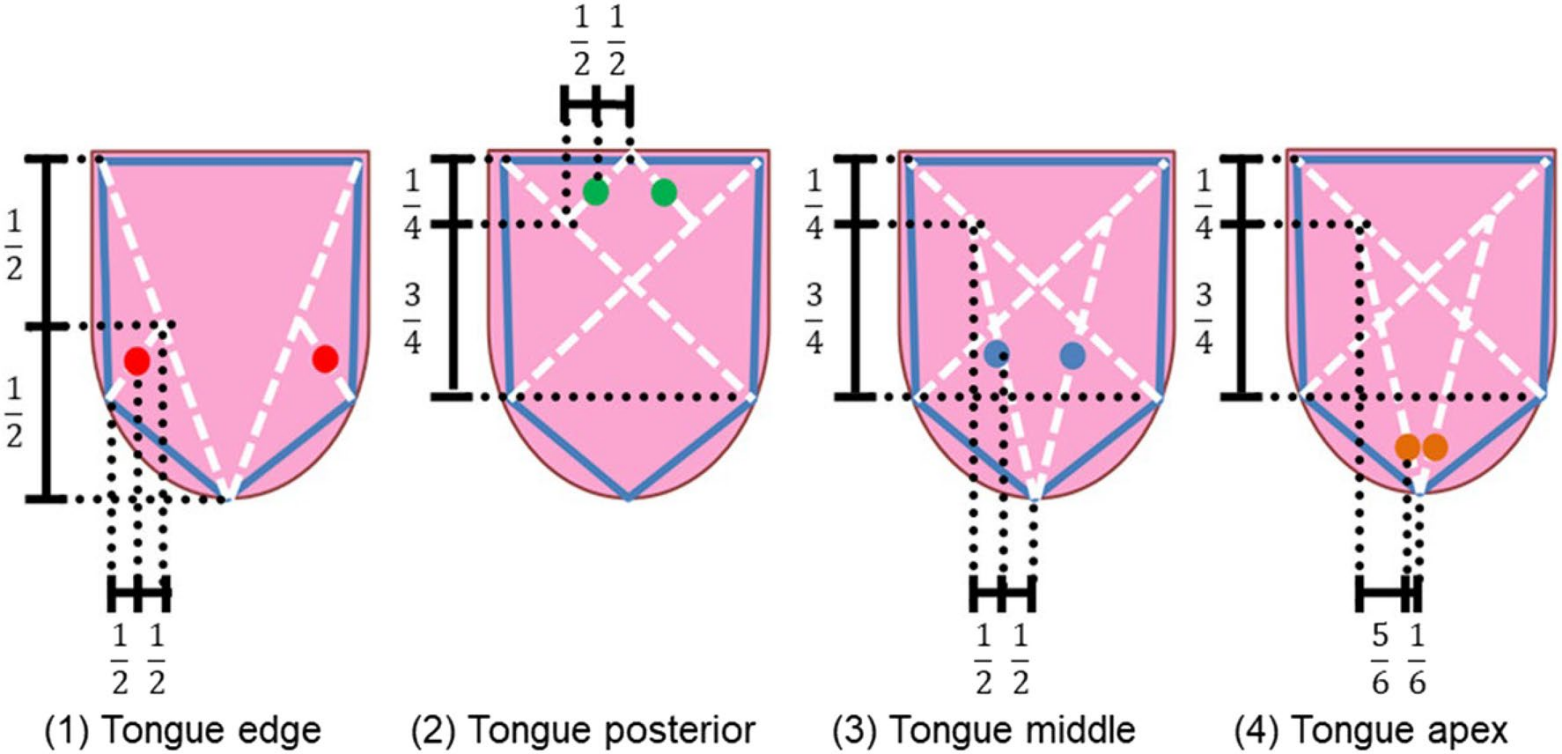
Definition of the areas. (1) Red corresponds to the tongue edge, (2) Green corresponds to the tongue posterior, (3) Blue corresponds to the tongue middle, and (4) Orange corresponds to the tongue apex.

Figure 2 shows the definition of the areas. As shown in the image, there is almost no tongue coating at the tongue apex (area 1), and the color of this area is similar to that of the tongue body. In contrast, the color of the tongue in the other three areas (areas 2–4) was a mixture of the color of the tongue coating and the color of the tongue body. This method of dividing the tongue into separate areas is traditionally used in tongue diagnosis.

### Statistical analysis

Statistical analysis data are expressed as number, or mean ± standard deviation (SD). We used the Student’s t-test and Mann–Whitney test for continuous variables. All statistical analyses were performed using scikit-learn, a machine learning library.

### Training and validation of the machine learning models

**Figure 3:**
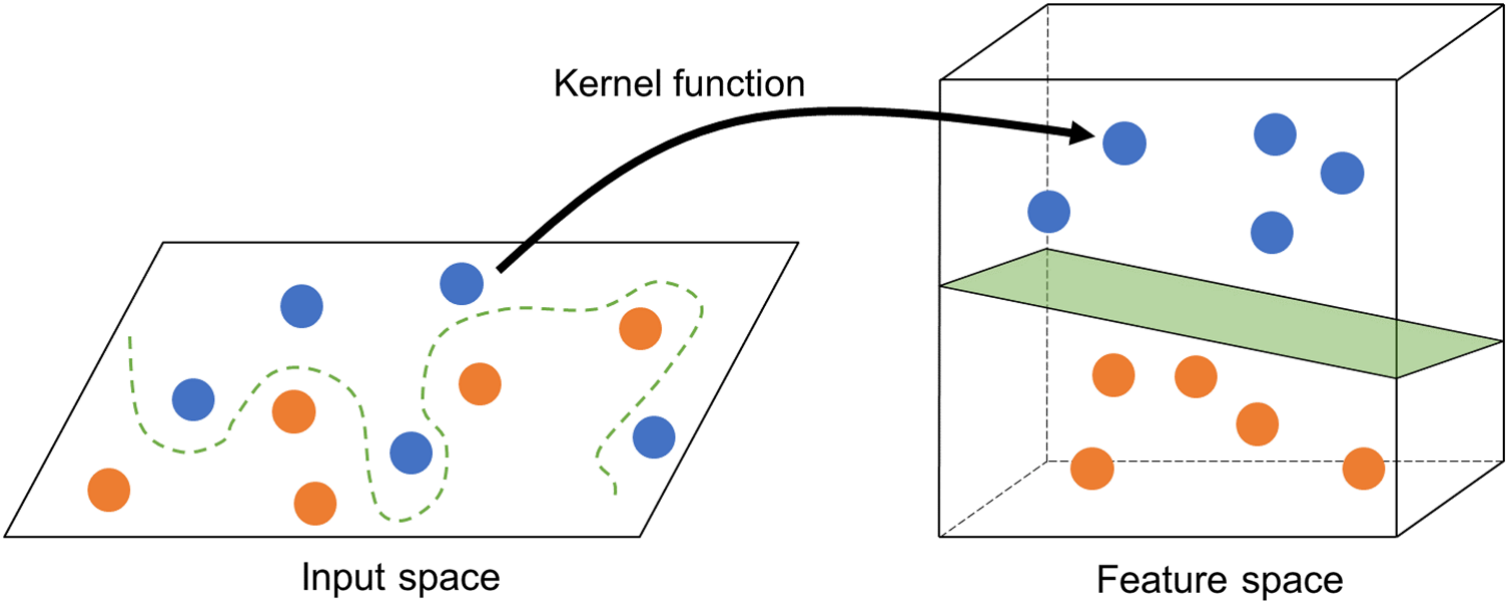
The basis of SVM classification.

**Figure 4:**
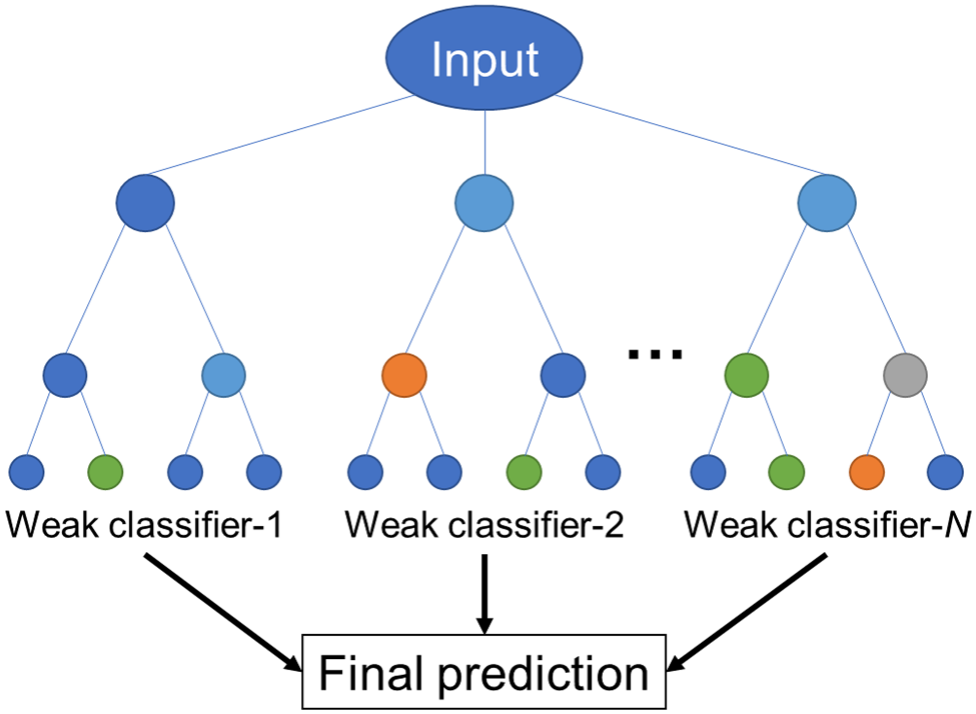
The basis of RF classification.

**Figure 5:**
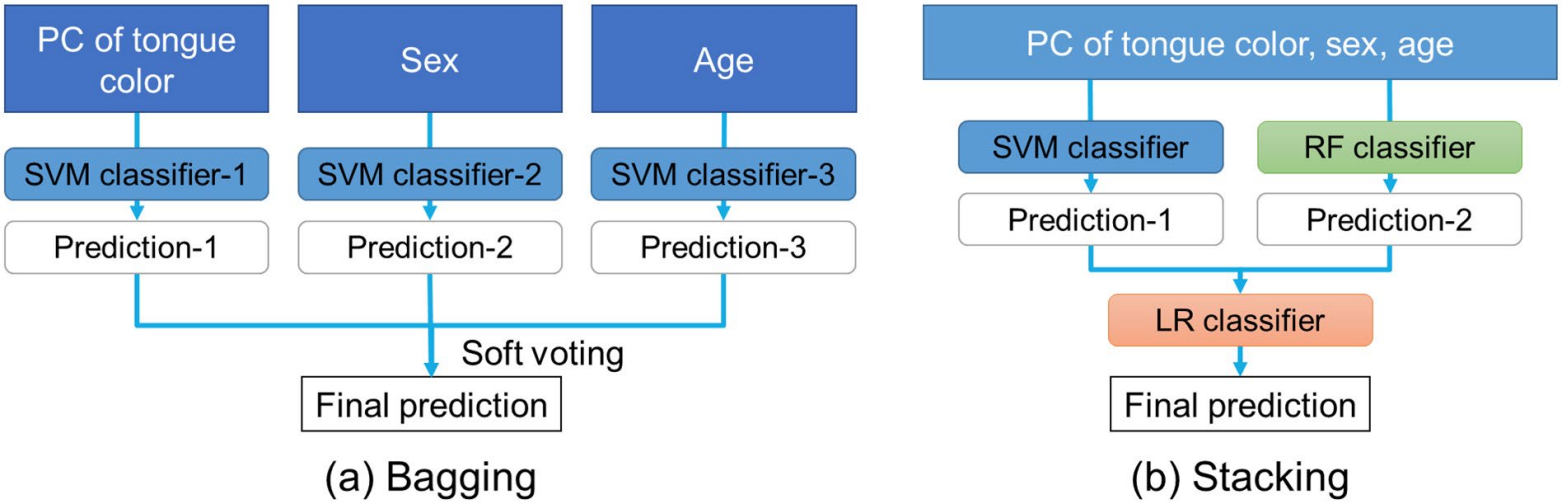
Ensemble of machine learning algorithms. (**a**) Bagging: Train several classifiers for each feature and combine the results by the soft voting. (**b**) Stacking: Use the predictions from multiple weak classifiers to train a model.

To clarify experimentally the possibility of diagnosing SS using the color of the tongue, we extracted information on the tongue color from the dataset and trained a machine learning classifier. For the first validation, three well-known algorithms: logistic regression (LR), support vector machine (SVM)^11^, and random forest (RF)^12^, were selected. LR is the oldest classification algorithm, and it uses a non-linear sigmoid function, which enables a slightly more complex division of images into classes compared to linear classification, which is based on a threshold. The classification of SVM is more non-linear than LR, because it uses the kernel method (see Fig. 3). In contrast, RF can learn multiple weak classifiers based on simple conditional branching and combine them to achieve a high-performance classification (see Fig. 4). Furthermore, to consider the effect of the ensemble learning by combining of these algorithms, we tried the use of the bagging method and the stacking method, shown in Fig. 5. The Bagging method in Fig. 5a uses three SVM models to predict SS by inputting the principal component scores of tongue color, gender, and age, respectively, and calculates the final prediction result by majority vote from the prediction results from the three models. The Stacking method in Fig. 5b uses one SVM model, one RF model, and one LR model. First, the SVM model outputs the prediction of SS with the input of the principal component score of tongue color, gender, and age. Next, the RF model also inputs the principal component scores of tongue color, gender, and age, and outputs the prediction of SS, and the LR model inputs the predictions of the SVM and RF models and calculates the final prediction.

Each parameter was optimized using a grid search, and the model was validated using stratified five-fold cross-validation. The search range of hyper-parameters for each algorithm is shown in Table 1. For the validation, first, the dataset was divided into five folds by aligning the classes' proportions, after which one of the folds was used for validation and the other four were used to train the model. The data was evaluated by averaging the scores of each fold. To reduce bias in the split, the average score was calculated by repeating the cross-validation 10 times.

**Table 1:** List of the hyper-parameter values.

Method	Hyper-parameter	Range of values
LR	Norm of the penalty	L2 norm
	Penalty parameter (C)	[10^−5^–10^−5^] in log_10_ step
SVM	Kernel	Radial Based Function (RBF)
	Penalty parameter (C)	[10^−5^–10^−5^] in log_10_ step
	Kernel coefficient (gamma)	[10^−5^–10^−5^] in log_10_ step
RF	The number of the tree	[1, 10, 50, 100]
	Maximum depth of the tree	[2, 3, 6, No limit]

The features input to the model were scaled to ensure that the mean and SD were 0 and 1, respectively. Then, dimensionality compression by principal component analysis was applied to exclude unimportant features with low variance and to aggregate the information. A principal component vector is calculated from the training samples and the 12 measured color values (L*, a*, b* values at four areas) are converted into three principal component scores by projecting them onto the first to third principal component vectors. This principal component scores are used as the input feature for machine learning. Lastly, to eliminate imbalance in the training dataset, we randomly oversampled the training cases in the training pipeline by using SMOTE^13^.

### Ethical consideration

The Ethics Committee at Tsurumi University School of Dental Medicine approved this study (approval number 244, Aug. 19, 2004 and approval number 521, Mar. 21, 2008) and all research was performed in accordance with the guidelines of the ethics committee. All patients provided written informed consent.

## Results

### Dataset

All human samples shown in Table 2 were seen at the outpatient clinic of Tsurumi University School of Dental Medicine. SS patients had typical symptoms of dry mouth such as difficulty in swallowing, impaired taste, or burning sensation of the tongue. Samples with stimulated values of < 2 g/ 2 min were diagnosed with dry mouth by Saxon test. They were diagnosed based on criteria proposed by the Japan Ministry of Health, Labor, and Welfare. These patients had not received glucocorticoids or immunosuppressive agents for at least 6 months prior to this examination. The samples were then divided into two different subgroups: SS Group with Saxon test of < 2 g/ 2 min, non-SS normal healthy individuals with Saxon test ≥ 2 g/ 2 min.

**Table 2:** Dataset used for the analysis.

	Non-SS patients (n = 41)	SS patients (n = 19)
Age (years)	66.3 ± 13.0	66.2 ± 8.9
Sex (male : female)	7 : 34	0 : 19
Saxon test (g)^a^	3.34 ± 2.22	1.72 ± 1.63

^a^There is a statistically significant difference in the Saxon test values (*p* < 0.01).

### Tongue color analysis

**Figure 6:**
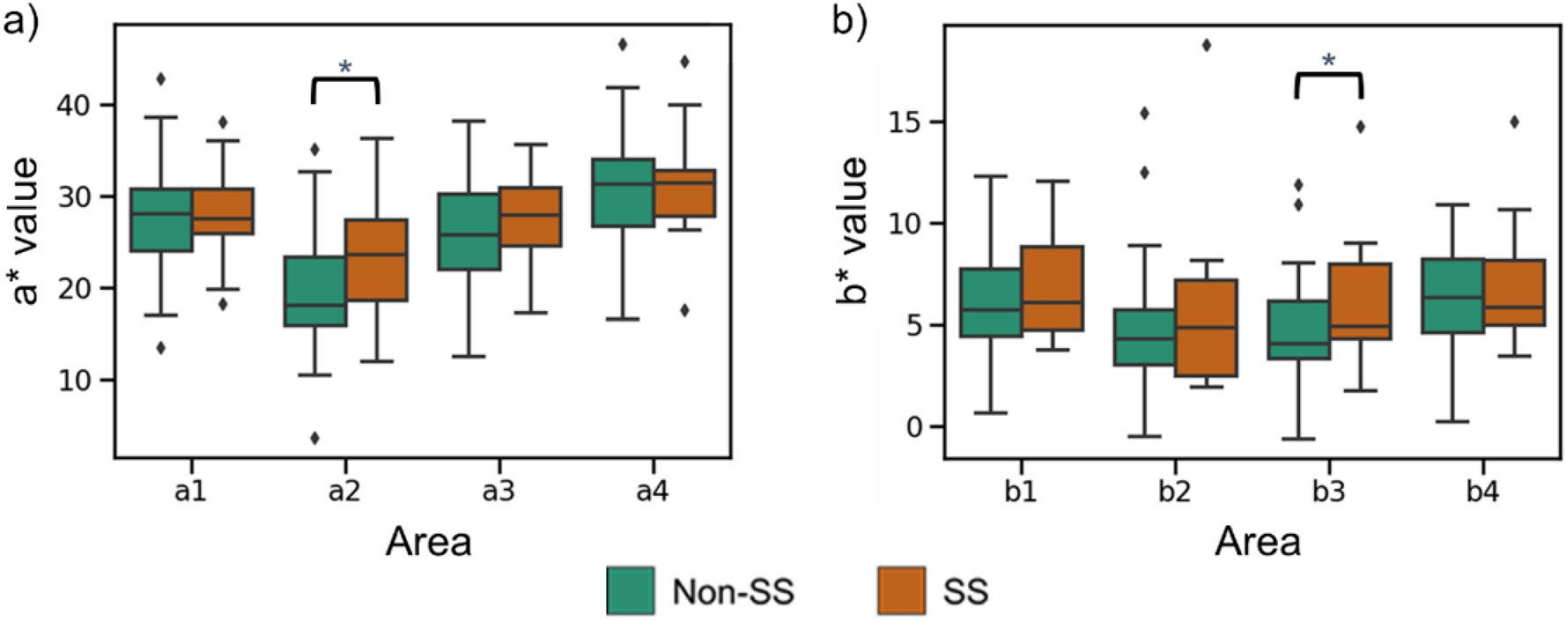
Comparison of the tongues of SS patients to those of non-SS patients. (**a**) a1-4 and (**b**) b1-4 indicate the a* and the b* of each tongue area defined in Fig. 2.

To quantitatively investigate the relationship between SS and tongue color, first, the CIE1976 L*a*b* values of the four areas were statistically analyzed. As shown in Table 3, there is a statistically significant difference in the a* values of area 2 (*p* < 0.05) and the b* values of area 3 (*p* < 0.05). The a* value represents the green–red component, with negative values toward green and positive values toward red. In addition, as shown in Fig. 6, the color of the tongue posterior of SS patients was more reddish than that of the tongue posterior of non-SS patients. Previous studies^2,14^ have suggested that the inflammation of the tongue of SS patients due to dry mouth causes a change in the color tone of the entire tongue to red. This hypothesis is consistent with the findings of this study, as the a* values of the tongue posterior of SS patients were significantly higher. In addition, there was a significant difference in b* values of the middle area, which may be attributed to the tongue coating. This is because patients with dry mouth often have a thick coating on their tongue due to the growth of oral bacteria.

**Table 3:** Tongue colors and result of the statistic test.

	Non-SS patients	SS patients	*p*-values
1L*	55.16 ± 10.84	55.20 ± 9.65	0.988
1a*	27.88 ± 5.71	28.28 ± 4.75	0.792
1b*	6.06 ± 2.47	6.77 ± 2.56	0.310
2L*	57.66 ± 8.36	53.68 ± 11.83	0.139
2a*	19.76 ± 6.23	23.79 ± 6.71	0.027^a^
2b*	4.59 ± 2.97	5.41 ± 3.95	0.372
3L*	59.64 ± 7.03	58.94 ± 8.42	0.736
3a*	25.82 ± 5.50	27.70 ± 5.40	0.220
3b*	4.64 ± 2.40	6.11 ± 2.89	0.042^b^
4L*	56.18 ± 9.78	56.56 ± 10.36	0.891
4a*	30.79 ± 6.41	31.33 ± 5.79	0.758
4b*	6.33 ± 2.48	6.88 ± 2.83	0.451

The numbers 1–4 correspond to the areas defined in Fig. 2.

^a,b^There is a statistically significant difference in the a* values of area 2 (*p* < 0.05) and the b* values of area 3 (*p* < 0.05).

### Machine learning result

**Figure 7:**
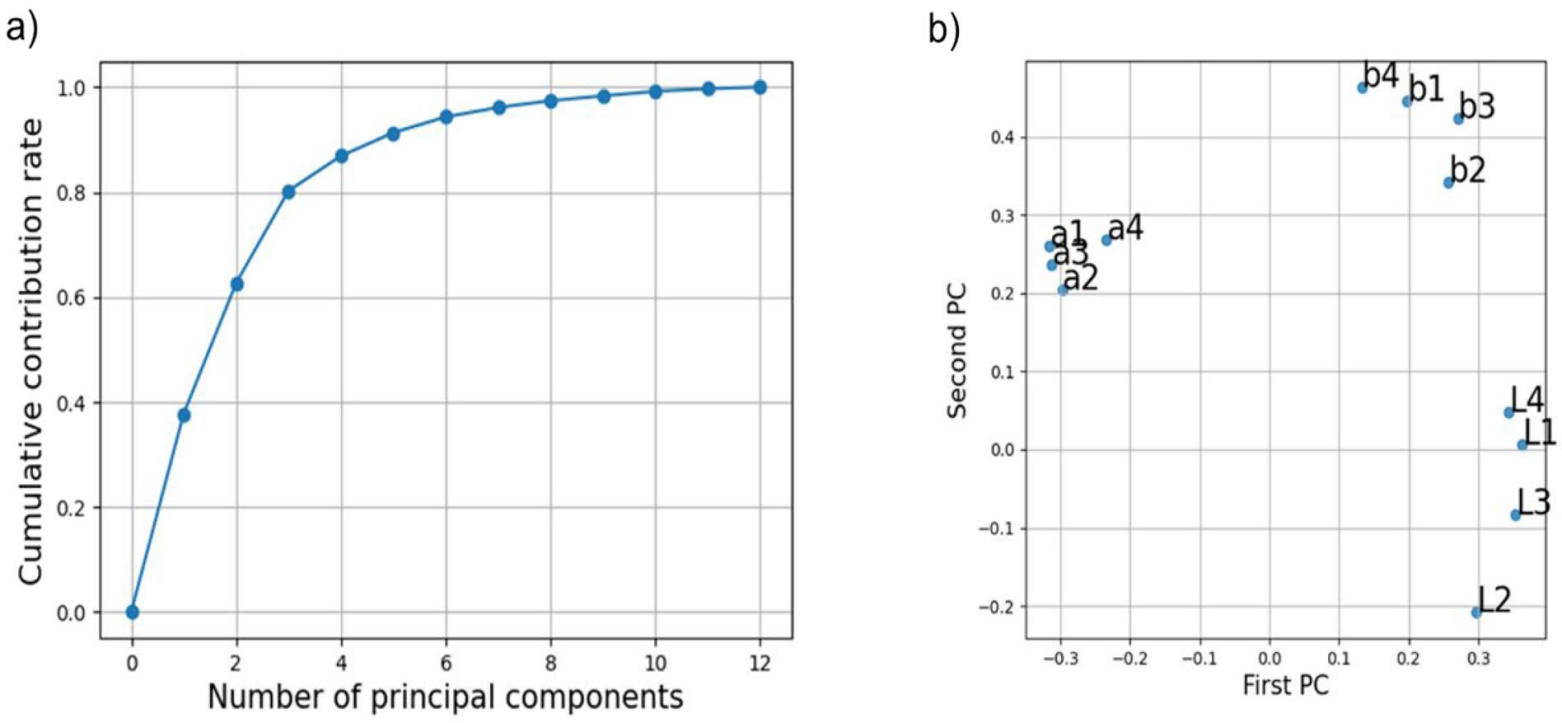
Result of the principal component analysis (PCA) of the tongue color. (**a**) Cumulative contribution rate of the principal components (PCs). (**b**) The distribution of factor loadings for the first and second principal components of the 12 color values is shown, where L, a, and b represent L*, a*, and b* values, and the numbers represent tongue regions (1:edge, 2:posterior, 3:middle, 4:apex).

First, the PCA was applied to the tongue color, and the calculated cumulative contribution rate of the principal components (PCs) are shown in Fig. 7a. The contribution rates of the first PC (PC1), the second PC (PC2), and the third PC (PC3) were 37.8%, 24.9%, and 17.6%, respectively. Thus, the cumulative contribution rate increased to 80.3% in PC3. The distribution of factor loadings for the PC1 and PC2 of the 12 color values is shown in Fig. 7b, where L, a, and b represent L*, a*, and b* values, and the numbers represent tongue regions (1:edge, 2:posterior, 3:middle, 4:apex). PC1 represents the a* and L* values, whereas PC2 represents the b* values, which resulted in a high cumulative contribution (62.7%) from PC1 and PC2. In addition, each L*a*b* value was treated almost equally regardless of the area; however, the L*a*b* value at area 2 was considered relatively higher or lower than those of the other areas. This may be attributed to the fact that the tongue posterior, indicated as area 2, tends to be covered with tongue coating and is different from the other areas.

To evaluate the data, we trained and compared LR, SVM and RF. As it is not desirable to use the area under the receiver operating characteristic or accuracy for evaluation when the number of each class is imbalanced, in this study, we used the area under the precision-recall curve (AP) by averaging the training results of each cross-validation (mAP). The results are shown in Table 4. Among the three classification algorithms, SVM exhibited the best performance, with an averagely high precision in terms of sensitivity. In contrast, LR exhibited the lowest specificity, which could be attributed to the fact that it predicted all cases as positive and failed to relate the input features to the classes. SVM outperformed RF on all features, indicating that it is more suitable for the prediction of SS. We also evaluated the stacking and the bagging, a type of ensemble learning, to investigate the effect of combining classifiers. Still, their performance was not as good as that of the SVM. The method proposed in this study achieved a high accuracy (71.3%) and specificity (78.1%) when the SVM was trained on the age and sex of the patient in addition to the PCs of tongue color. The dimensionality reduction effectively improved the performance of this method.

**Table 4:** Comparison of the mean scores of the cross-validation experiments.

Model	Features	Accuracy	Sensitivity	Specificity	Precision	F score	Kappa score	mAP
LR	Saxon	0.665	0.735	0.633	0.500	0.580	0.354	0.670
	Tongue color	0.330	0.985	0.026	0.319	0.481	0.007	0.514
	PC of Tongue color	0.317	1.000	0.000	0.317	0.480	0.000	0.562
	PC of Tongue color, sex, age	0.432	1.000	0.169	0.361	0.529	0.119	0.560
SVM	Saxon	0.675	0.730	0.650	0.512	0.584	0.334	0.670
	Tongue color	0.472	0.895	0.281	0.374	0.518	0.134	0.510
	PC of Tongue color	0.625	0.567	0.652	0.454	0.487	0.208	0.580
	PC of Tongue color, sex, age	**0.713**^a^	0.575	0.781	0.591	0.546	0.354	**0.664**^**b**^
RF	Saxon	0.598	0.643	0.578	0.433	0.496	0.197	0.601
	Tongue color	0.370	0.980	0.089	0.334	0.496	0.048	0.502
	PC of Tongue color	0.317	1.000	0.000	0.317	0.480	0.000	0.568
	PC of Tongue color, sex, age	0.615	0.692	0.580	0.448	0.529	0.239	0.609
Bagging	PC of Tongue color, sex, age	0.573	0.450	0.621	0.361	0.382	0.070	0.515
Stacking	PC of Tongue color, sex, age	0.652	0.568	0.695	0.510	0.499	0.257	0.647

^a,b^SVM trained using PC of Tongue color, sex, and age showed the best accuracy and AP.

Significant values are in [bold].

## Discussion

The accuracy of color conversion from RGB to L*a*b* values was verified by calculating the root mean square error (RMSE) using a sample of 128 colors that are different from the 24 colors used for calibration and are similar to tongue and skin colors. The results showed that the errors for L*, a*, and b* were 5.5, 2.1, and 3.9, respectively. Compared to the standard deviation of each color value in Table 3, the L* and a* color conversion errors were found to be smaller than the variation in the dataset, while the b* error was larger than the variation in the dataset. Improving the accuracy of b* color conversion is an issue for the future.

Since the dataset size used in this study is small (60), classical machine learning models such as LR, SVM, and RF were used instead of complex models such as deep learning. The results show that no overfitting occurred because the test data, which is separated from training data, also showed high prediction performance.

In this study, to clarify the relationship between the tongue and SS, we obtained and analyzed the images of the tongues of SS patients. The color of the tongue was extracted and converted into CIE1976L*a*b* space by dividing it into four areas based on Kampo medicine. The results revealed that there was a statistically significant difference in the a* value at the tongue posterior and in the b* value at the middle of the tongue of SS and non-SS patients. This suggests that the appearance of the tongue was changed in SS patients owing to tongue inflammation and increased tongue coatings. To further investigate the relationship between SS and the tongue, we trained a machine learning classifier to diagnose SS based on the color of the tongue. In this experiment, the best scores were obtained using PCA, and the results revealed that SVM was the most suitable classification algorithm for predicting SS, followed by RF, and finally LR. The lowest performance of LR was attributed to its significantly lower specificity, with LR outputting positive results in all cases. This can be attributed to the inability to associate features of the input data with negative classes. On the other hand, RF is considered to be advantageous for classification tasks with high dimensional input data. However, the input data used in this study was 12-dimensional before dimensionality compression and 3-dimensional after, and the number of dimensions of the input data was not that high. As shown in Fig. 7a, the cumulative contribution ratio is 99% up to 9 dimensions. Therefore, it is considered that the characteristics of RF could not be utilized. Although the size of the dataset was limited in this study, the performance was improved by combining the dimensionality reduction using PCA and SVM, which is robust to small numbers of data. In addition, we found that the adding age of the patient further improved the diagnostic performance of this method to a level comparable to that of a classifier trained using the Saxon test, whereas the efficacy of sex information was not able to be evaluated due to lack of male patient in positive cases. A related study^10^ shows a high prediction performance with AUC = 0.84. Although the AUC could not be calculated in this study and was compared with the mAP value, the prediction performance of the study^10^ is considered to be higher than that of this study. However, while the related studies require primary care data recorded regularly, the method proposed in this study is very simple, requiring only a single photograph of the tongue and age information. We consider that this study is the only method that can contribute to the early detection of SS patients widely because it can be applied to various situations such as physical examinations and mass screening tests. The statistical and machine-learning methods used in this study revealed that there was a change in the appearance of the tongue of SS patients, indicating its promising potential for the clinical diagnosis of SS.

A report^15^ showed that Sjögren's syndrome has a significant sex ratio, with more than 90% of patients being female. Therefore, it can be considered that gender is important feature to predict SS. The fact that the SS-positive data set in this study does not include male data raises concerns about bias in the evaluation of the prediction accuracy of the machine learning model. This point needs to be verified by expanding the dataset in the future. Furthermore, the number of positive cases is less than half of the number of negative cases. In other words, there is a general tendency for the output to be biased toward the class with the majority of cases. However, in this study, despite the large number of negative cases, the sensitivity of the positive detection is very high. Therefore, the effect of data imbalance is small. We plan to further investigate this point by increasing the number of cases in the future.

In subsequent studies, we will be investigating the possibility of distinguishing SS patients from non-SS patients in a group of patients with dry mouth. In addition, we will be reaching out to universities and clinics to train more complex models, such as convolutional neural networks, by increasing the number of cases and comparing the results to that of our method.

## Data Availability

The datasets generated during and/or analyzed during the current study are available from the corresponding author on reasonable request.
